# Differential associations of emotional and physical domains of the MacNew Heart with changes in 6-min walking test

**DOI:** 10.1007/s11136-022-03247-3

**Published:** 2022-10-11

**Authors:** Lena Jellestad, Vera G. Meier, Walter Bierbauer, Tania Bermudez, Bianca Auschra, Moritz P. Günther, Urte Scholz, Roland von Känel, Matthias Hermann, Sebastian Euler

**Affiliations:** 1 Department of Consultation-Liaison Psychiatry and Psychosomatic Medicine, University Hospital Zurich, Ramistrasse 100, 8091 Zurich, Switzerland; 2 Department of Psychology, University of Zurich, Zurich, Switzerland; 3 Department of Cardiology, University Heart Center, University Hospital Zurich, Zurich, Switzerland; 4 University Research Priority Area Dynamics of Healthy Aging, University of Zurich, Zurich, Switzerland

**Keywords:** HRQOL, Cardiac rehabilitation, Exercise capacity, 6MWT

## Abstract

**Aims:**

Cardiac rehabilitation (CR), a key component of secondary prevention in cardiac patients, contributes fundamentally to improved cardiovascular health outcomes. Health-related quality of life (HRQOL) represents a widely employed outcome measure in CR, yet, its predictive properties on exercise capacity change during CR are poorly understood. Aim of this study was to examine the association between baseline HRQOL and its subdomains on improvement of exercise capacity during CR.

**Methods:**

Study participants were 13,717 inpatients of six Swiss CR clinics from 2012 to 2018. We measured HRQOL at admission to CR with the MacNew Heart (MNH) questionnaire and exercise capacity at admission and discharge using the six minutes walking test (6MWT). Following factorial analyses, we performed univariate and multivariate analyses to test the predictive properties of baseline global HRQOL and its domains for improvement in exercise capacity, adjusting for demographic and clinical characteristics.

**Results:**

Mean improvement in 6MWT was 114 m (SD = 90), achieved after 17.4 days (SD = 5.5). Lower emotional HRQOL (*b* = 7.85, *p* =  < .001, 95% CI [− 5.67, 10.03]) and higher physical HRQOL (*b* =  − 5.23, *p* < .001, 95% CI [− 6.56, − 3.90]) were associated with less improvement in the 6MWT. Global MNH and social HRQOL showed no association with exercise capacity improvement.

**Conclusion:**

Patients entering CR with low emotional and high physical HRQOL are at risk for a lower gain in exercise capacity during CR. Global MNH alone does not provide a reliable assessment of HRQOL; thus a focus on specific domains of HRQOL is needed.

**Supplementary Information:**

The online version contains supplementary material available at 10.1007/s11136-022-03247-3.

## Introduction

Cardiovascular diseases (CVD) are the leading cause of global disease burden, requiring increasing health care expenditures [1]. Cardiac rehabilitation (CR) is strongly recommended by international guidelines [2], contributing to improved cardiovascular health outcomes by reducing cardiovascular mortality, morbidity and hospitalization [3]. CR comprises three-phases including the acute inpatient phase (phase I), the subacute and reconditioning phase (phase II), and the long term maintenance period (phase III). In Switzerland, phase II inpatient CR programs last three weeks and encompass a set of core components defined by the Swiss Working Group for Cardiovascular Prevention, Rehabilitation, and Sports Cardiology (www.scprs.ch). These components include exercise-based training, smoking, and nutritional counseling, as well as psychosocial and psychoeducational interventions. Quality measurements of cardiac rehabilitation clinics are implemented and monitored by the Swiss National Association for the Development of Quality in Hospitals and Clinics (ANQ; https://www.anq.ch/en).

Considering the major impact of psychosocial factors on the development and course of CVD—ranking third among the modifiable risk factors for CVD, after hyperlipidemia and smoking [4]—the importance of integrated psychosocial interventions is highly emphasized. A widely employed screening tool and outcome measure of therapeutic interventions in cardiac patients is health-related quality of life (HRQOL) [5], a multidimensional construct of the patients’ perceived health. Poor HRQOL has previously been identified as an independent predictor of survival, (re-)hospitalization [6], mortality [7], and cardiovascular morbidity in cardiac patients [8]. Although evidence on the relationship between HRQOL and exercise capacity is emerging, until now, HRQOL has primarily been recognized as an outcome variable in CR [9, 10]. To date, and only recently, two smaller studies have examined the predictive potential of non-disease specific HRQOL on improvement of metabolic equivalents of tasks (METs) as a marker of exercise capacity in CR. While one found no significant association [11], the second one detected a positive association between physical HRQOL and improvement in exercise capacity [12].

The aim of our study was to investigate the association between HRQOL at admission to CR and exercise capacity change during CR with a disease-specific and thus more suitable HRQOL questionnaire in a large sample from 6 Swiss rehabilitation clinics. We first aimed to explore the association between global HRQOL at admission to CR and CR outcome, assessed by a change in exercise capacity during CR using the six minutes walking test (6MWT) as a proxy of CR effectiveness. The second aim was to explore the predictive value of individual HRQOL domains on CR outcome. We hypothesized that better global HRQOL and its individual domains at admission to CR would be associated with greater improvement in exercise capacity from admission to discharge from CR. Covering the relevant domains of HRQOL- emotional, physical, and social indices- we applied the MacNew Heart (MNH) questionnaire as a well-known, disease-specific and validated outcome measure of HRQOL in cardiac patients [13].

## Materials and methods

### Study design and participants

**Fig. 1:**
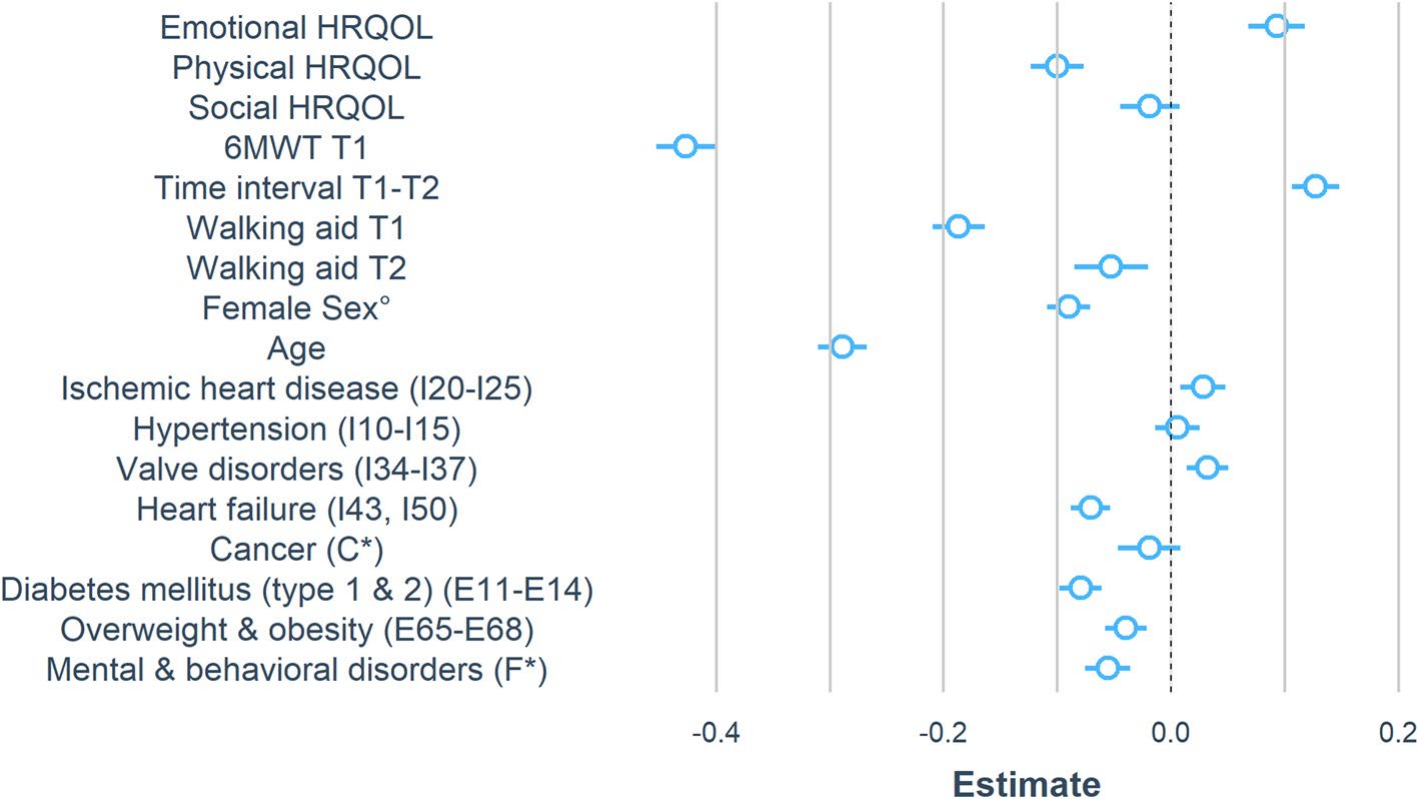
Vertical dashed line depicts the null effect, horizontal blue lines show the confidence interval of *β*, the circle marks *β* itself. *Indicating chapter of the ICD-10, ºvs. male sex

This study used a single group pre- and post-test design. Participants comprised cardiac patients of inpatient phase II CR programs of six Swiss inpatient rehabilitation clinics between November 2012 and December 2018. Data of *N* = 18,459 records of Swiss in-patient CR clinics prospectively assessed as part of a national quality assessment program (Swiss National Association for the Development of Quality in Hospitals and Clinics—ANQ; https://www.anq.ch/en/) were considered eligible for this study. Of those, patients with a minimum of seven and a maximum of 56 days of inpatient treatment were included. Following a study by Bierbauer et al. [14], a minimum of seven days was defined, assuming that changes in exercise capacity, measured by the 6MWT, can be attributed to a CR program at the earliest after one week of treatment. We excluded all patients with missing data on the 6MWT. We also excluded all patients who were rated with 0 m in the 6MWT but who did not perform the 6MWT at enrollment and/or discharge, unless the reason given for non-participation was 'being too sick'. The latter cases were included as true zeros. All other reasons (e.g., ‘rejection by patient’) defined by a categorical variable and recorded by clinic staff were excluded from analysis. After screening for multivariate outliers using the Mahalanobis distance [15], *N* = 2 patients were additionally removed from the data set. The study was approved by the Cantonal Ethics Committee of the Canton Zurich; REQ-2019–00,291 (Fig. 1).

### Measures

#### Exercise capacity

The 6MWT was applied as a validated and widely used objective measure of exercise capacity in patients with coronary heart diseases [16]. The test measures the distance (in meters) a patient can walk on a flat, hard surface within a fixed time period of 6 min. As the 6MWT is designed to assess the submaximal functional capacity it is also suitable for physically limited cardiac patients. Since most activities of daily life are performed at a submaximal level, this test better reflects physical functioning in everyday life than tests assessing maximum functional capacity (e.g., spiroergometry) [16]. The walking distance was recorded with enrollment in CR (T1) and toward discharge (T2) from CR. Changes in exercise capacity were estimated by the difference in walking distance between the two measurement points subtracting the distance walked at TI from that at T2. Thus, higher scores indicate improvement. In cardiac patients, a change of + 45 m in distance is considered to be clinically relevant [17].

### Health-related quality of life (HRQOL)

HRQOL was measured using the German adaptation of the MacNew Heart (MNH) HRQOL Questionnaire, a self-report questionnaire for the disease-specific QoL of cardiovascular patients [18]. It is a modified version of the Quality of Life after Myocardial Infarction [QLMI] Questionnaire, which was initially developed as an instrument to assess quality of life in patients with myocardial infarction undergoing CR [19]. The MNH consists of 27 items, referring to an emotional, physical, and social domain of HRQOL. Scores in any domain range from a maximum of 7 (high HRQOL) and a minimum of 1 (poor HRQOL), which can be calculated to a global score as the average of all item scores per domain. The German version of the MNH was validated on inpatient cardiac patients from Austria and Switzerland [18]. A score change of 0.5 points is considered the minimum clinically relevant change in the global score and any domain in MNH [20]. Item 27 on sexual activity was excluded from analysis due to frequent missings, which is an accepted procedure [13].

### Statistical analysis

Statistical analyses were performed using R (R Version 3.6.1, RStudio Version 1.2.5019) [21]. The primary outcome variable was the change in meters in the 6MWT (∆distance of 6MWT) from CR entry to CR discharge (T2–T1), with higher values indicating greater improvement in exercise capacity.

In our preliminary analyses, the factor structure of Höfer et al. [18] did not provide an acceptable fit to our data. This finding was in line with previous studies that showed the questionable nature of the proposed structure [22, 23] and suggested performing secondary analyses for the most appropriate grouping of items [22]. As such, using two different random samples of the total sample we first performed an exploratory factor analysis (EFA) of the three domains of the MNH HRQOL (emotional, physical, social), followed by a confirmatory factor analysis (CFA) using the Full-Information-Maximum-Likelihood (FIML) method. We hereby followed an established approach, which has already been applied in a previous validation analysis on the MNH [24]. The CFA showed an acceptable fit for the allocation of items to the domains of HRQoL (Table 1 of Supplemental for the factor structure of HRQOL).

**Table 1:** Characteristics of 13,717 study participants

Characteristics	*n* (%)	*M* (SD)
*Age (in years)*		69.1 (11.8)
*Sex*		
Female	4′985 (36.3%)	
Male	8′734 (63.7%)	
*Insurance status*		
Standard	9′525 (69.4%)	
(Semi-)private	4′194 (30.6%)	
*Duration of stay (in days)*		20.2 (5.3)
*Rehabilitation clinic*		
Clinic A	3′749 (27.3%)	
Clinic B	3′131 (22.8%)	
Clinic C	1′562 (11.4%)	
Clinic D	2′368 (17.3%)	
Clinic E	1′890 (13.8%)	
Clinic F	1′019 (7.4%)	
*Clinical characteristics (according to ICD-10)*		
Hypertension (I10–I15)	7′399 (53.9%)	
Ischemic heart disease (I20–I25)	7′606 (55.4%)	
Valve disorders (I34–I37)	4′144 (30.2%)	
Heart failure (I43, I50)	1′680 (12.3%)	
Diabetes mellitus (type 1 and 2) (E11–E14)	2′422 (17.7%)	
Cancer (C*)	559 (4.1%)	
Overweight, obesity, other hyperalimentation (E65–E68)	875 (6.4%)	
Mental and behavioral disorders (F*)	2′424 (17.8%)	
*6-min walking test*		
T1 (m)		291 (141)
T2 (m)		405 (151)
Change from T1–T2 (m)		114 (90)
Time interval T1–T2 (d)		17.4 (5.5)
Walking aid T1	3′337 (24.3%)	
Walking aid T2	2′720 (19.8%)	

*6MWT* 6-min walking test, *T1* baseline score at enrollment in CR, *T2* score at discharge of CR, *M* Mean, *SD* standard deviation, *m* meters, *d* days, *Indicating chapter of the ICD-10

Based on our factor analyses, we performed a univariate FIML linear regression analysis to reveal the association of global HRQOL at T1 and the change in exercise capacity (T2–T1) and a multivariate linear regression analyses to assess the associations of the different domains of HRQOL on ∆distance of 6MWT (T2–T1). In both models, we controlled for the following rehabilitation-related covariates: 6MWT at T1 in order to adjust for the initial level of exercise capacity, interval in days between T1 and T2; use of a walking aid; age; sex; health insurance status (standard and (semi)private); and origin of data (i.e., rehabilitation clinic). In addition, we controlled for the following main and secondary diagnoses according the International Statistical Classification of Disease (ICD)-10: hypertension (I10-I15), ischemic heart disease (I20-I25), valve disorders (I34-I37), heart failure (I43, I50), diabetes mellitus (type 1 and 2) (E11-E14), cancer (chapter two of the ICD-10), overweight, obesity, and other hyperalimentation (E65-E68), as well as mental and behavioral disorders (chapter V of the ICD-10). Diagnoses were entered as dummy variables.

## Results

### Baseline characteristics

Of the original *N* = 18′459 patients the final sample comprised *n* = 13′717 patients after exclusion, whose characteristics are depicted in Table 1. Men accounted for two-thirds of the study population, the average age of participants was 70 years. The mean duration of CR in our sample corresponds to the generally recommended three weeks of inpatient CR treatment of cardiac patients. Hypertension and ischemic heart disease were the leading cardiac diagnoses of the sample and were present in approximately half of the participants. About every 6th patient was diagnosed with a psychiatric condition. The mean improvement in exercise capacity (6MWT) over the course of CR across all patients was clinically relevant and averaged 114 m (SD = 90).

### Global HRQoL and exercise capacity

The mean score of global HRQOL in our sample was 4.93 (SD = 1.08). In a first step we performed a univariate linear regression analysis with all control variables (baseline 6MWT, time interval between T1 and T2, use of a walking aid, age, sex, insurance status, medical diagnoses, rehabilitation clinic) to analyze the association of global HRQOL with exercise capacity and these control variables. The regression of global HRQOL on change in exercise capacity and the above mentioned control variables are displayed in Table 2, using an FIML estimation to take into account missing data. Adjusted for all control variables global HRQOL was not significantly associated with an improvement in exercise capacity (*b* =  − 0.71, *p* = 0.425).

**Table 2:** Results of multiple linear regression on global HRQOL and change in exercise capacity

Predictors	Coefficient (b)	95% CI of b	*p*	β
*Global HRQOL*	− 0.26	[− 2.46, 1.04]	.425	− 0.01
*6MWT T1*	− 0.30	[− 0.32, − 0.29]	< .001	− 0.43
Time interval T1–T2 (d)	1.74	[1.48, 2.01]	< .001	0.13
Walking aid T1	− 41.35	[− 45.40, − 37.29]	< .001	− 0.19
Walking aid T2	− 18.18	[− 24.45 − 11.90]	< .001	− 0.05
*Age*	− 2.04	[− 2.17, − 1.90]	< .001	− 0.29
*Female Sex*^*º*^	− 19.94	[− 22.82, − 17.50]	< .001	− 0.10
*Insurance status*	11.22	[8.04, 14.38]	< .001	0.05
*Rehabilitation clinic*				
Clinic A	14.35	[8.56, 20.14]	< .001	0.06
Clinic B	7.54	[1.74, 13.35]	.010	0.07
Clinic C	7.07	[0.46, 13.68]	.024	0.03
Clinic D	52.12	[46.08, 58.17]	< .001	0.23
Clinic E	24.59	[18.18, 30.00]	< .001	0.10
*Clinical characteristics (according to ICD-10)*				
Hypertension (I10–I15)	− 0.56	[− 3.56, 2.43]	.713	0.00
Ischemic heart disease (I20-I25)	7.17	[4.24, 10.09]	< .001	0.03
Valve disorders (I34–I37)	10.43	[7.31, 13.55]	< .001	0.04
Heart failure (I43, I50)		[− 25.54, − 17.30]	< .001	− 0.07
Diabetes mellitus (type 1 and 2) (E11–E14)	− 21.02	[− 24.64, − 17.40]	< .001	− 0.08
Cancer (C*)	− 1.85	[− 8.62, 4.92]	.593	− 0.02
Overweight, obesity, other hyperalimentation (E65–E68)	− 13.98	[− 19.57, − 8.38]	< .001	− 0.04
Mental and behavioral disorders (F*)	− 13.67	[− 17.31, − 10.04]	< .001	− 0.07

*HRQOL* health-related quality of life, *6MWT* 6-min walking test, *T1* baseline score at enrollment in CR, *T2* score at discharge of CR, *β* standardized regression weights, *CI* Confidence interval, *d* days, *Indicating chapter of the ICD-10, º vs. male sex

### Domains of HRQoL and exercise capacity

The mean score of emotional HRQOL in the sample was highest (*M* = 5.26, SD = 1.14), followed by the score of social HRQOL (*M* = 5.07, SD = 1.18) and physical HRQOL (*M* = 4.66, SD = 1.53). Table 3 shows the multivariate regression of the three domains of HRQOL on change in exercise capacity; all three domains were entered simultaneously into the model. Adjusted for the identical control variables as in the univariate linear regression for global HRQOL, we found higher emotional HRQOL at T1 to be significantly associated with greater improvement in the 6MWT between T1 and T2 (*b* = 7.85, *p* < 0.001, 95% CI [− 5.67, 10.03]). In other words, lower emotional HRQOL was associated with less improvement in exercise capacity. Higher physical HRQOL at T1 was significantly associated with less improvement in the 6MWT between T1 and T2 (*b* =  − 5.23, *p* < 0.001, 95% CI [− 6.56, − 3.90]). This indicates that patients who started out with better physical HRQOL had less improvement in exercise capacity. Social HRQOL at T1 was not significantly associated with an improvement in the 6MWT between T1 and T2 (*b* =  − 1.27, *p* = 0.22, 95% CI [− 3.23, 0.76]). See Table 3 for the detailed results of the multivariate regression analysis.

**Table 3:** Results of multivariate regression analysis on domains of HRQOL and change in exercise capacity

Predictors	Coefficient (b)	95% CI of b	*p*	β
*Emotional HRQOL*	7.85	[− 5.67, 10.03]	< .001	0.10
*Physical HRQOL*	− 5.23	[− 6.56, − 3.90]	< .001	− 0.10
*Social HRQOL*	− 1.27	[− 3.23, 0.76]	.219	− 0.02
*6MWT T1*	− 0.30	[− 0.32, − 0.29]	< .001	− 0.48
Time interval T1–T2 (d)	1.66	[1.39, 1.92]	< .001	0.10
Walking aid T1	− 40.79	[− 44.85, − 36.74]	< .001	− 0.20
Walking aid T2	− 18.79	[− 25.16, 12.43]	< .001	− 0.05
*Age*	− 2.00	[− 2.14, − 1.87]	< .001	− 0.26
*Female Sex*^*º*^	− 18.02	[− 20.92, − 15.11]	< .001	− 0.10
*Insurance status*	10.04	[6.87, 13.20]	< .001	0.05
*Rehabilitation clinic*				
Clinic A	15.62	[9.84, 21.42]	< .001	0.08
Clinic B	12.89	[6.07, 19.70]	< .001	0.06
Clinic C	8.93	[2.34, 15.54]	.004	0.03
Clinic D	51.76	[45.70, 57.82]	< .001	0.22
Clinic E	26.85	[20.43, 33.27]	< .001	0.10
*Clinical characteristics*				
Hypertension (I10–I15)	0.14	[− 2.85, 3.13]	.927	0.00
Ischemic heart disease (I20–I25)	7.33	[4.41, 10.25]	< .001	0.04
Valve disorders (I34–I37)	9.59	[6.47, 12.70]	< .001	0.05
Heart failure (I43, I50)	− 20.66	[− 24.77, − 16.55]	< .001	− 0.08
Diabetes mellitus (type 1 and 2) (E11–E14)	− 20.39	[− 24.01, − 16.78]	< .001	− 0.09
Cancer (C*)	− 1.43	[− 8.27, 5.40]	.682	− 0.00
Overweight, obesity, other hyperalimentation (E65–E68)	− 14.43	[− 20.01, − 8.85]	< .001	− 0.04
Mental and behavioral disorders (F*)	− 10.97	[− 14.63, − 7.39]	< .001	− 0.05

*HRQOL* health-related quality of life, *6MWT* 6-min walking test, *T1* baseline score at enrollment in CR; *T2* score at discharge of CR; *β* standardized regression estimates, *CI* Confidence interval, *d* days; *Indicating chapter of the ICD-10, ^º^vs. male sex

## Discussion

This is the first study to analyze the predictive value of baseline global HRQOL and its individual domains on CR outcome assessed by an improvement in exercise capacity using the 6MWT. We employed a multi-center approach including a large sample of *n* = 13′717 patients across 6 Swiss inpatient CR clinics.

To the best of our knowledge, to date there are only two studies, which recently investigated the predictive potential of baseline HRQOL on change in exercise capacity in CR using metabolic equivalents (METs) as a measure of exercise capacity [11, 12]. While METs and 6MWT have a strong positive correlation and are both valid measures of exercise capacity [25], the much smaller sample of these studies and the use of a non-disease specific HRQOL questionnaire (short form-36 (SF-36)) limits their explanatory power.

Our findings confirm the initial assumption of a positive association of emotional HRQOL with greater improvement in the 6MWT during CR. In turn, this means that lower emotional HRQOL might be a potential risk factor for less improvement of exercise capacity throughout CR. As such, the construct of emotional HRQOL might represent an important determinant of CR outcome. Encompassing items on feeling ‘frustrated,’ ‘worthless,’ ‘tearful,’ ‘frightened,’ emotional HRQOL has been linked to affective disorders due to partial conceptual overlap (e.g., anxiety and depression) [26]. Affective symptoms (e.g., anxiety and depression) have similarly been identified as relevant determinants of worse CR outcome [27]. Further, the perception of lower emotional health [28] and negative affectivity [29] have previously been linked to adherence as a relevant psychosocial risk factor for poor CR outcome, increasing the risk of non-completion and non-adherence. These mental conditions on admission to CR could thus potentially translate into worse adherence throughout CR and consequently contribute to a poorer CR outcome. Considering this, an early and thorough identification of vulnerable patients directly after (or even before) admission to CR may enable an optimized support to patients in need and could substantially contribute to an improved CR outcome. Not only do CR patients themselves value psychosocial support as an integral component of the CR program [30], but psychologically based interventions have been advocated in improving CR outcome. Enhanced psychological care [31] and integrated stepped-care programs embedded within CR [32], targeted to improve care for CR patients with depression and anxiety have correspondingly received increased attention in recent years. While significant effects in the reduction of affective symptoms were achieved compared to usual care [32], limitations became evident in the integration of these specialized psychological services into the clinical context of CR [31]. However, patients in the subsyndromal range of affective symptomatology, as captured by emotional HRQOL, could be easily overlooked in the allocation to specialized care. Thus, by focusing solely on individuals with affective disorders on CR entry, opportunities may be missed to identify and support emotionally impaired individuals at risk for less improvement in exercise capacity. Utilizing lower threshold assessments might thus help to identify patients already with subsyndromal emotional impairments. These patients could particularly benefit from closer supervision of their exercise program or individually tailored high intensity interval training during CR by physical therapists. Clearly, before firm recommendations can be made, such interventions should be tested in randomized controlled trials for their efficacy and safety in the subgroup of patients with low emotional HRQOL. Also, it has to be confirmed in future studies, whether the effect of low emotional wellbeing on adherence truly mediates the effect on CR outcome.

Since evidence indicates patients to be especially motivated and determined to make behavioral and lifestyle changes during inpatient setting [33], intensive psychosocial care in this crucial phase is particularly relevant. Yet, psychological care within CR is often provided by nurses, who consider it essential for patient recovery, but often enough encounter barriers in providing support sufficiently due to time and resource constraints [30]. Multidisciplinary approaches integrating psychological interventions provided by mental healthcare professionals may thus better address patients’ needs [32] and help to mitigate frequent organizational constraints in providing optimal support in the future. As mental health outcome is also predicted by CR waiting time and a delay in commencing CR significantly impacts anxiety and depression [34] and possibly emotional HRQOL, keeping the wait time to CR as short as possible is also highly important to promote better emotional HRQOL at CR entry and consecutively the best possible CR outcome.

Contrary to the initial assumption, our findings indicate a negative association of physical HRQOL and change in exercise capacity during CR: Higher physical HRQOL at admission to CR was linked to a lower improvement in exercise capacity. While from a clinical perspective it is encouraging that inversely patients with low physical QoL benefit more from CR, patients who feel only mildly physically burdened by the symptoms of their disease on entering CR, reflected in higher values of physical HRQOL, may possibly be less motivated to participate in CR. An alternative explanation may be a ceiling effect in that people who already experience relatively good physical health may benefit less from the CR measures and thus improve less in the 6MWT as a physical outcome variable. Moreover, prior findings have linked physical QOL to the construct of illness perception, defined as expectations and beliefs about ones illness- a negative illness perception being correlated with lower physical QOL [35]. In turn, a positive illness perception has been demonstrated to predict lower adherence to CR procedures [36]. Consequently, in patients with higher physical HRQOL, resultant poorer adherence could thus ultimately contribute to the poorer improvement in exercise capacity.

We found no impact of social HRQOL on improvement in exercise capacity during CR. Evidence on the benefit of social support in the framework of CR is contradictory. While our results are in line with findings by Husak et al. [37], they contradict earlier evidence of a positive correlation between perceived social support (as a construct of social HRQOL) and effectiveness of CR [37, 38]. Previous validity analyses on the German version of the MNHs factor structure have already pointed to a difficulty of interpreting the social domain due to poor construct validity [39]. Also, the inpatient survey context of this study may account for some of the differences in findings. Questions on social HRQOL might be less relevant in inpatient treatment and answers might rather reflect the perceived support by the treatment team than by personal caregivers. This assumed, a different construct of social HRQOL may have been covered by the underlying design of our study; the anticipated positive correlations between social HRQOL and change in exercise capacity may be more evident in an outpatient setting.

Whereas abundant evidence links higher global HRQOL to a positive course of cardiac diseases [6–8], global HRQOL did not predict CR outcome in terms of exercise capacity in our study. This might be related to the internal structure of the MNH (with a cross-dimensional global index based on the average of the emotional, social, and physical MNH scores). Since the physical and emotional domain of HRQOL showed opposite effects on exercise capacity in our results, their individual effect might mutually neutralize each another, thus amounting to the missing effect of global HRQOL on exercise capacity. In line with this and given the complex and multidimensional concept of HRQOL, arguments have also been raised against the use of a global HRQOL score, favoring to consider individual HRQOL dimensions separately [40].

## Strengths and limitations

Our investigation of multidimensional HRQOL as a possible determinant for the effectiveness of CR fills an important gap in the existing literature. Although it is well known that psychosocial factors play a crucial role in the effectiveness of CR [41], evidence on the prognostic properties of HRQOL on exercise capacity has so far been missing. The thorough factor-analytical approach to the MNH items prior to the main analysis allowed the theory and data-driven identification of the most appropriate definition of the HRQOL domains for the present data set, which formed a solid basis for the subsequent main analyses.

Further major strengths of the present study are the large sample size, the multi-center approach, and the inclusion of a wide spectrum of cardiac diagnoses, which substantiate the representativeness of the sample.

Yet, some limitations have to be acknowledged. Our large sample size enabled us to thoroughly investigate the predictive properties of HRQOL on exercise capacity, however, the effects found were small. Moreover, given the multidimensionality of the quality of life concept, many different HRQOL survey instruments have been applied in the past, also in cardiovascular patients. This wide range of available instruments represents a challenge in research on this topic in general and can also contribute to the divergent findings across studies. Moreover, the factor structure of the MNH may affect the generalizability of existing evidence, especially, since score composition also varies by language [39].

Further, due to the correlational design, no causal effect of CR can be inferred. The observed changes in exercise capacity may also be driven by factors other than HRQOL. Training intensity, for instance, was identified as the most critical predictor of exercise capacity at discharge of CR [42], a variable we could not deduce from the present data set. While in several European countries, inpatient phase II CR services are provided, this is not the case on a global level, where phase II CR is often only provided in an outpatient setting. This might limit generalizability of our results. In light of this, replication of our results in outpatient settings would be desirable in future studies.

## Conclusion

The results of our study highlight a need for an adequate and reliable assessment of HRQOL that is not limited to an evaluation of the global HRQOL. Rather, the imperative of focusing on specific domains of HRQOL must be emphasized. In our study, emotional and physical HRQOL emerged as potentially valuable determinants of CR outcome. Particular focus should be directed toward patients with lower emotional HRQOL, who are at risk for lower gain in exercise capacity during CR. As a broad and dimensional construct, emotional HRQOL allows for a particularly applicable assessment of vulnerable patients even with subsyndromal emotional impairments being at risk for worse CR outcome. Efforts should therefore be directed at early identification of these patients in the CR admission assessment. Only then, early and targeted interventions might allow to modulate CR outcome and to mitigate unfavorable impacts of poor emotional HRQOL. Future research efforts should be aimed at developing such interventions for an early support of patients with poorer emotional HRQOL and examine their impact on CR outcome. Also, the potential influence of individual domains of HRQOL on adherence as an overarching determinant of CR outcome should be considered.

## Supplementary Information

Below is the link to the electronic supplementary material.

Supplementary file1 (DOCX 13 kb)

## Data Availability

All data used in this study are deposited and part of the national quality assessment program (Swiss National Association for the Development of Quality in Hospitals and Clinics—ANQ; https://www.anq.ch/en/).

## References

[1] World Health Organization. (WHO). (2017). Cardiovascular disease (CVDs). Health topics. https://www.hoint/news-room/fact-sheet/detail/cardiovascular-disease-(cvds).

[2] Price KJ, Gordon BA, Bird SR, Benson AC (2016). A review of guidelines for cardiac rehabilitation exercise programmes: Is there an international consensus?. European journal of preventive cardiology..

[3] Anderson L, Oldridge N, Thompson DR, Zwisler AD, Rees K, Martin N (2016). Exercise-based cardiac rehabilitation for coronary heart disease: Cochrane systematic review and meta-analysis. Journal of the American College of Cardiology..

[4] Yusuf S, Hawken S, Ounpuu S, Dans T, Avezum A, Lanas F (2004). Effect of potentially modifiable risk factors associated with myocardial infarction in 52 countries (the INTERHEART study): Case-control study. Lancet (London, England)..

[5] Rumsfeld JS, Alexander KP, Goff DC, Graham MM, Ho PM, Masoudi FA (2013). Cardiovascular health: The importance of measuring patient-reported health status: A scientific statement from the American Heart Association. Circulation.

[6] Alla F, Briançon S, Guillemin F, Juillière Y, Mertès PM, Villemot JP (2002). Self-rating of quality of life provides additional prognostic information in heart failure. Insights into the EPICAL study. European Journal of Heart Failure.

[7] Hofer S, Benzer W, Oldridge N (2014). Change in health-related quality of life in patients with coronary artery disease predicts 4-year mortality. International Journal of Cardiology.

[8] Dixon T, Lim LLY, Heller RF (2001). Quality of life: An index for identifying high-risk cardiac patients. Journal of Clinical Epidemiology..

[9] Francis T, Kabboul N, Rac V, Mitsakakis N, Pechlivanoglou P, Bielecki J (2019). The effect of cardiac rehabilitation on health-related quality of life in patients with coronary artery disease: A meta-analysis. The Canadian Journal of Cardiology.

[10] Candelaria D, Randall S, Ladak L, Gallagher R (2020). Health-related quality of life and exercise-based cardiac rehabilitation in contemporary acute coronary syndrome patients: A systematic review and meta-analysis. Quality of life Research: An International Journal of Quality of Life Aspects of Treatment, Care and Rehabilitation.

[11] Gathright EC, Goldstein CM, Loucks EB, Busch AM, Stabile L, Wu WC (2019). Examination of clinical and psychosocial determinants of exercise capacity change in cardiac rehabilitation. Heart & Lung: The Journal of Critical Care.

[12] Abu-Haniyeh A, Shah NP, Wu Y, Cho L, Ahmed HM (2018). Predictors of cardiorespiratory fitness improvement in phase II cardiac rehabilitation. Clinical Cardiology.

[13] Höfer S, Lim L, Guyatt G, Oldridge N (2004). The MacNew Heart Disease health-related quality of life instrument: A summary. Health and Quality of Life Outcomes.

[14] Bierbauer W, Scholz U, Bermudez T, Debeer D, Coch M, Fleisch-Silvestri R (2020). Improvements in exercise capacity of older adults during cardiac rehabilitation. European Journal of Preventive Cardiology.

[15] Yuan K-H, Zhong X (2008). 8 Outliers, leverage observations, and influential cases in factor analysis: Using robust procedures to minimize their effect. Sociological Methodology.

[16] Guyatt GH, Sullivan MJ, Thompson PJ, Fallen EL, Pugsley SO, Taylor DW (1985). The 6-minute walk: A new measure of exercise capacity in patients with chronic heart failure. Canadian Medical Association Journal.

[17] Shoemaker MJ, Curtis AB, Vangsnes E, Dickinson MG (2012). Triangulating clinically meaningful change in the six-minute walk test in individuals with chronic heart failure: A systematic review. Cardiopulmonary Physical Therapy Journal..

[18] Hofer S, Benzer W, Schussler G, von Steinbuchel N, Oldridge NB (2003). Health-related quality of life in patients with coronary artery disease treated for angina: Validity and reliability of German translations of two specific questionnaires. Quality of Life Research: An International Journal of Quality of Life Aspects of Treatment, Care and Rehabilitation.

[19] Oldridge N, Guyatt G, Jones N, Crowe J, Singer J, Feeny D (1991). Effects on quality of life with comprehensive rehabilitation after acute myocardial infarction. The American Journal of Cardiology.

[20] Dixon T, Lim LL, Oldridge NB (2002). The MacNew heart disease health-related quality of life instrument: Reference data for users. Quality of Life Research: An International Journal of Quality of Life Aspects of Treatment, Care and Rehabilitation.

[21] Team RC (2014). R: A Language and Environment for Statistical Computing. R Foundation for Statistical Computing.

[22] Dempster M, Donnelly M, O'Loughlin C (2004). The validity of the MacNew quality of life in heart disease questionnaire. Health and Quality of Life Outcomes.

[23] Alphin S, Höfer S, Perk J, Slørdahl S, Zwisler AO, Oldridge N (2015). The MacNew heart disease health-related quality of life questionnaire: A scandinavian validation study. Social Indicators Research.

[24] Seneviwickrama KLMD, Samaranayake DBDL, Fonseka P, Galappaththy GNL, Höfer S, Oldridge NB (2016). Psychometric evaluation of the Sinhalese version of MacNew heart disease health related quality of life questionnaire in patients with stable angina. Health and Quality of Life Outcomes.

[25] Saba MA, Goharpey S, Attarbashi Moghadam B, Salehi R, Nejatian M (2021). Correlation between the 6-min walk test and exercise tolerance test in cardiac rehabilitation after coronary artery bypass grafting: A cross-sectional study. Cardiology and Therapy.

[26] Stafford L, Berk M, Reddy P, Jackson HJ (2007). Comorbid depression and health-related quality of life in patients with coronary artery disease. Journal of Psychosomatic Research.

[27] Rao A, Zecchin R, Newton PJ, Phillips JL, DiGiacomo M, Denniss AR (2020). The prevalence and impact of depression and anxiety in cardiac rehabilitation: A longitudinal cohort study. European Journal of Preventive Cardiology.

[28] Hershberger PJ, Robertson KB, Markert RJ (1999). Personality and appointment–keeping adherence in cardiac rehabilitation. Journal of Cardiopulmonary Rehabilitation.

[29] Meyer F, von Känel R, Saner H, Schmid J-P, Stauber S (2014). Positive affect moderates the effect of negative affect on cardiovascular disease-related hospitalizations and all-cause mortality after cardiac rehabilitation. European Journal of Preventive Cardiology.

[30] Turner KM, Winder R, Campbell JL, Richards DA, Gandhi M, Dickens CM (2017). Patients' and nurses' views on providing psychological support within cardiac rehabilitation programmes: A qualitative study. British Medical Journal Open.

[31] Richards SH, Dickens C, Anderson R, Richards DA, Taylor RS, Ukoumunne OC (2018). Assessing the effectiveness of Enhanced Psychological Care for patients with depressive symptoms attending cardiac rehabilitation compared with treatment as usual (CADENCE): A pilot cluster randomised controlled trial. Trials.

[32] Child A, Sanders J, Sigel P, Hunter MS (2010). Meeting the psychological needs of cardiac patients: An integrated stepped-care approach within a cardiac rehabilitation setting. British Journal of Cardiology.

[33] Abreu A (2019). In-hospital psychological intervention in cardiac rehabilitation following acute coronary syndrome: Brief is better than nothing. Portuguese Journal of Cardiology: An Official Journal of the Portuguese Society of Cardiology.

[34] Sumner J, Böhnke JR, Doherty P (2018). Does service timing matter for psychological outcomes in cardiac rehabilitation? Insights from the national audit of cardiac rehabilitation. European Journal of Preventive Cardiology.

[35] Alsén P, Brink E, Persson L-O, Brändström Y, Karlson BW (2010). Illness perceptions after myocardial infarction: Relations to fatigue, emotional distress, and health-related quality of life. Journal of Cardiovascular Nursing..

[36] Whitmarsh A, Koutantji M, Sidell K (2003). Illness perceptions, mood and coping in predicting attendance at cardiac rehabilitation. British journal of health psychology..

[37] Husak L, Krumholz HM, Lin ZQ, Kasl SV, Mattera JA, Roumanis SA (2004). Social support as a predictor of participation in cardiac rehabilitation after coronary artery bypass graft surgery. Journal of Cardiopulmonary Rehabilitation.

[38] Shen BJ, McCreary CP, Myers HF (2004). Independent and mediated contributions of personality, coping, social support, and depressive symptoms to physical functioning outcome among patients in cardiac rehabilitation. Journal of Behavioral Medicine.

[39] Gramm L, Farin E, Jaeckel WH (2012). Psychometric properties of the German version of the MacNew heart disease health-related quality of life questionnaire. Health and Quality of Life Outcomes.

[40] Höfer S, Benzer W, Brandt D, Laimer H, Schmid P, Bernardo A (2004). MacNew Heart Disease Lebensqualitätsfragebogen nach Herzinfarkt. Zeitschrift für Klinische Psychologie und Psychotherapie.

[41] Pogosova N, Saner H, Pedersen SS, Cupples ME, McGee H, Höfer S (2015). Psychosocial aspects in cardiac rehabilitation: From theory to practice. A position paper from the Cardiac Rehabilitation Section of the European Association of Cardiovascular Prevention and Rehabilitation of the European Society of Cardiology. European Journal of Preventive Cardiology.

[42] Uddin J, Zwisler AD, Lewinter C, Moniruzzaman M, Lund K, Tang LH (2016). Predictors of exercise capacity following exercise-based rehabilitation in patients with coronary heart disease and heart failure: A meta-regression analysis. European Journal of Preventive Cardiology.

