# Supplementary material for: Differential associations of emotional and physical domains of the MacNew Heart with changes in 6-min walking test
Source: Qual Life Res. 2022 Oct 11;32(2):615–24. doi: 10.1007/s11136-022-03247-3 (PMC9911504; doi:10.1007/s11136-022-03247-3)
Supplement: Supplementary file 1 — Supplementary file1 (DOCX 13 kb) [file 11136_2022_3247_MOESM1_ESM.docx]

**Table 1. Allocation of MNH- items to domains of HRQOL**

| **Domains of HRQOL** | **MNH- Items** |
| --- | --- |
| Emotional | 1 ,2 ,3 , 4, 5, 7, 8, 10, 18 |
| Physical | 17, 20, 26 |
| Social | 11, 13, 23 |

HRQOL = Health Related Quality of Life; MNH = MacNew Heart.
